# Predischarge Prediction of Readmission After Cytoreductive Surgery and Hyperthermic Intraperitoneal Chemotherapy: Derivation and Validation of a Risk Prediction Score

**DOI:** 10.1245/s10434-020-09547-7

**Published:** 2021-01-23

**Authors:** Caroline J. Rieser, Lauren B. Hall, Eliza Kang, Amer H. Zureikat, Matthew P. Holtzman, James F. Pingpank, David L. Bartlett, M. Haroon A. Choudry

**Affiliations:** 1 Division of Surgical Oncology, Koch Regional Perfusion Center, University of Pittsburgh, Pittsburgh, PA USA; 2 AHN Cancer Institute, Allegheny Health Network, Pittsburgh, PA USA

## Abstract

**Background:**

Ninety-day hospital readmission rates following cytoreductive surgery and hyperthermic intraperitoneal chemotherapy (CRS/HIPEC) range from 20 to 40%.

**Objective:**

The aim of this study was to develop and validate a simple score to predict readmissions following CRS/HIPEC.

**Study Design:**

Using a prospectively maintained database, we retrospectively reviewed clinicopathologic, perioperative, and day-of-discharge data for patients undergoing CRS/HIPEC for peritoneal surface malignancies between 2010 and 2018. In-hospital mortalities and discharges to hospice were excluded. Multivariate logistic regression was utilized to identify predictors of unplanned readmission, with three-quarters of the sample randomly selected as the derivation cohort and one-quarter as the validation cohort. Using regression coefficient-based scoring methods, we developed a weighted 7-factor, 10-point predictive score for risk of readmission.

**Results:**

Overall, 1068 eligible discharges were analyzed; 379 patients were readmitted within 90 days (35.5%). Seven factors were associated with readmission: stoma creation, Peritoneal Cancer Index score ≥ 15, hyponatremia, in-hospital major complication, preoperative chemotherapy, anemia, and discharge to nursing home. In the validation cohort, 25 patients (9.2%) were categorized as high risk for readmission, with a predicted rate of readmission of 69.3% and an observed rate of 76.0%. The score had fair discrimination (area under the curve 0.70) and good calibration (Hosmer–Lemeshow goodness-of-fit *p*-value of 0.77).

**Conclusion:**

Our proposed risk score, easily obtainable on day of discharge, distinguishes patients at high risk for readmission over 90 days following CRS/HIPEC. This score has the potential to target high-risk individuals for intensive follow-up and other interventions.

**Supplementary Information:**

The online version of this article (10.1245/s10434-020-09547-7) contains supplementary material, which is available to authorized users.

Cytoreductive surgery and hyperthermic intraperitoneal chemotherapy (CRS/HIPEC) is an aggressive and complex treatment for patients with disseminated peritoneal malignancies. Due to the high rates of morbidity, postoperative care is often as complex as the surgery itself.^1^,^2^ As a consequence, readmissions following CRS/HIPEC are frequent, occurring after 20–40% of discharges.^1^,^3^ With increasing regionalization and fragmentation of surgical care, up to 20% of surgical oncology readmissions may occur at non-index hospitals, with associated increased risk for major complications.^3^–^5^ In addition to impacting patient quality of life and cost of care, readmissions and associated complications may both drive worse cancer-specific survival.^1^,^4^,^6^

While many interventions are currently being proposed to reduce readmissions following complex oncologic surgery, there is no validated tool for predicting readmission for patients undergoing CRS/HIPEC to guide application and track outcomes for such interventions. Current surgery-specific risk prediction tools heavily weight complications and cancer diagnoses, rendering them inappropriate for use in the CRS/HIPEC population, where all patients have disseminated cancer and upwards of 60% of patients experience at least one postoperative complication and 26% experience a major complication.^1^,^2^,^7^,^8^

To efficiently target patients for interventions aimed at decreasing readmission, we need to first reliably identify a high-risk population prior to discharge. This would allow providers to appropriately direct future efforts aimed at improving patient care and oncologic outcomes. The aim of this study was to examine the drivers of readmission among patients undergoing CRS/HIPEC and derive and validate a simple score to predict the risk of readmission at discharge.

## Methods

### Study Design and Population

A retrospective cohort study was conducted of all patients undergoing CRS/HIPEC at a single-center, quarternary surgical oncology hospital that is part of a 40-hospital healthcare system, from 1 January 2010 through 31 December 2018, using a prospectively maintained database. Patients were excluded from the analysis if they died during, or were discharged to hospice after, index hospitalization. Planned readmissions were excluded from this analysis. This study was approved by the Institutional Review Board at the University of Pittsburgh (IRB 19010278).

### Study Outcome

Our study outcome of interest was readmission following hospitalization for CRS/HIPEC. We defined readmission as unplanned hospitalization of any duration within 90 days of discharge.^9^ These included readmissions to both index and non-index facilities. Admissions to skilled nursing facilities or long-term acute care facilities following discharge were not counted as readmissions.^10^

A secondary outcome of interest was readmission due to surgical quality of care. This was chosen as this subset of readmissions would be theoretically targetable for perioperative interventions to prevent readmissions. Readmissions were labeled as related to surgical quality of care based on the presence of International Classification of Diseases (ICD) codes for readmission diagnoses established by Mull and colleagues^11^ via a Delphi consensus process. Readmissions with diagnoses of infection, sepsis, pneumonia, hemorrhage/hematoma, anemia, ostomy complications, acute renal failure, failure to thrive, fluid/electrolyte disorders, or venous thromboembolism (VTE) were considered to be associated with surgical quality. Patients could have more than one cause for readmission if more than one relevant diagnosis code was present on readmission.

### Predictor Variables

Data were collected from several sources for this analysis, including preoperative evaluation, perioperative record, postoperative course, and day-of-discharge characteristics. The following patient characteristics were examined: sex, body mass index (BMI, kg/m^2^), age-adjusted Charlson–Deyo Comorbidity Index (AA-CCI), smoking status, repeat CRS/HIPEC, and American Society of Anesthesiologists’ (ASA) physical status score. In addition, the following oncologic factors were also examined: primary histology, Eastern Cooperative Oncology Group (ECOG) score, preoperative chemotherapy receipt, and number of preoperative chemotherapy cycles.

The following operative details were examined: prior surgery score (PSS; 0 = no surgery or biopsy only; 1 = exploratory laparotomy, only 1 region dissected; 2 = exploratory laparotomy with resections, two to five regions dissected; and 3 = extensive previous cytoreduction, more than five regions dissected), Peritoneal Cancer Index (PCI) score as determined at the time of operation, operative length (hours), estimated blood loss (EBL, mL), intraoperative transfusion, number of visceral resections, number of anastomoses, and completeness of cytoreduction (CC) score.

The following hospitalization details were collected: length of stay (days), in-hospital complication (any), comprehensive complication index score (CCI total), major (Clavien–Dindo grade III or higher) complication rate, in-hospital anastomotic leak, in-hospital VTE event, postoperative transfusion, percutaneous drain placement, in-hospital need for total parenteral nutrition (TPN), return to the intensive care unit (ICU) during index admission, and return to the operating room (OR) during index admission.

Several discharge factors were examined: discharge with stoma (ileostomy or colostomy), day-of-discharge hemoglobin (g/dL), white blood cell count (10^9^ cells/L) and sodium (mEq/L) levels, discharge on antibiotics, therapeutic anticoagulation, TPN, number of discharge medications, and discharge to a skilled nursing facility

All variables were chosen *a priori* based on previously published literature.^3^,^7^,^8^,^10^,^12^–^14^

### Missing Data

Missingness was quantified for all predictor variables (electronic supplementary Table 1), was minimal, and was <5%, with the exception of the number of pre-CRS/HIPEC chemotherapy cycles (28%). We used a multiple imputation approach using 10 imputed datasets via chained multiple imputations to handle missing data.^15^

### Statistical Methods

The cohort was randomized into a derivation (75%) set and a separate validation (25%) set. Descriptive statistics were reported for the whole cohort and the derivation cohort by readmission status. Continuous data were reported as mean and standard deviation (SD) when normally distributed, and median with interquartile range (IQR) when non-normally distributed. Categorical data were reported as frequencies and percentages.

All perioperative variables were evaluated for possible significance in predicting readmission via univariate logistic regression in the derivation cohort. Variables with a *p*-value < 0.30 on univariate analysis were included in an initial backwards stepwise elimination multivariable logistic regression model. Using backwards elimination, variables were sequentially removed from the model until all remaining predictors had a *p*-value < 0.05.^10^ For ease of interpretation and application into a simple score, in the final model continuous variables were converted into dichotomous variables, with cut-offs determined by a minimum *p*-value approach.^16^ Using regression coefficient-based scoring methods, the multivariable model was then converted into an integer-based score.^17^

The probability of readmission was then examined by risk score within the derivation cohort and patients were categorized into groups based on predicted risk for readmission (low, moderate, and high), establishing score cut-offs for each group.^10^ The risk score was then applied to the validation cohort. Model calibration was assessed by examining the observed and expected rates of readmission within the validation cohort and via the Hosmer–Lemeshow goodness-of-fit test. Model discrimination was evaluated within the validation cohort by examining the receiver operating characteristic (ROC) curve and calculating the area under the ROC curve (AUC).

Model performance was evaluated between individual components and the full model by examination of the AUC. The Brier score was also used to assess overall model accuracy and performance, with possible scores ranging from 0 to 1 and lower scores indicating better performance. Spiegelhalterʼs z‐statistic was used to test the significance of the Brier score; a *p*-value < 0.05 indicates poor calibration.

A two-sided *p*-value < 0.05 was considered significant for all tests. The data were analyzed using STATA 15 (StataCorp LLC, College Station, TX, USA).

## Results

### Patient Cohort

**Fig. 1:**
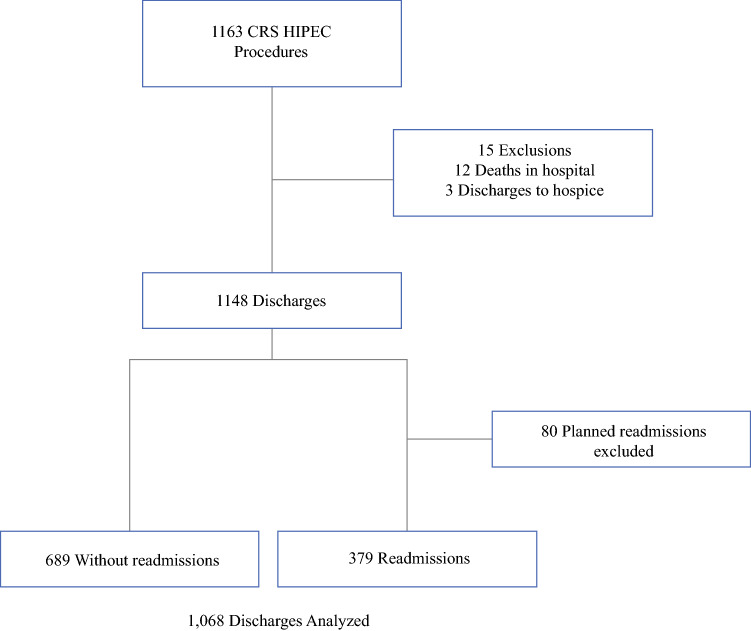
Study diagram. From 2010 to 2018, 1163 CRS/HIPEC procedures were performed at our institution. Fifteen patients were excluded from the analysis due to death or discharge to hospice. Among 1148 discharges, 80 had planned readmissions for secondary procedures and were excluded from the analysis, resulting in 1068 discharges being analyzed, with 379 instances (35.5%) of readmission. *CRS* cytoreductive surgery, *HIPEC* hyperthermic intraperitoneal chemotherapy

During the study period, 1163 CRS/HIPEC procedures were performed (Fig. 1). Fifteen patients were excluded due to death during hospitalization or discharge to hospice. Eighty patients underwent planned readmission for ileostomy takedown within 90 days of discharge and were excluded from this analysis. Overall, 962 patients accounted for 1068 eligible discharges following CRS/HIPEC. All 1068 encounters were included in the final analysis. Of these, 379 patients (35.5%) were readmitted within 90 days, with a median time to readmission of 12 days (IQR 6–22). Of these readmissions, 288 (76.0%) readmissions were categorized as related to quality of surgical care (Table 1). The primary reasons for readmission were infectious complications and fluid or electrolyte issues; 134 (35.4%) readmissions were labeled as failure to thrive.

**Table 1:** Readmission details

Variable	*n* (%)
30-day readmission rate	320 (30.0)
90-day readmission rate	379 (35.5)
Non-index hospital readmission	109 (28.8)
Time to readmission, days [median (IQR)]	12 (6–22)
Admission labeled as failure to thrive	134 (35.4)
Admission related to surgical care	288 (76.0)
Infection	162 (56.3)
Fluid status or kidney function	152 (52.8)
Ostomy care or complications	45 (15.6)
Deep vein thrombosis or pulmonary embolism	23 (8.0)

The 1068 encounters were randomly divided into a derivation cohort (*n* = 796) and a validation cohort (*n* = 272). Table 2 demonstrates the baseline characteristics for the overall and derivation cohorts. Median patient age was 56 years and approximately half of the cohort was male; 138 (13%) patients underwent repeat CRS/HIPEC. The primary tumor histologies were appendiceal (46%) and colorectal (29%), and the majority of patients (60%) underwent preoperative chemotherapy, with a median number of six cycles (IQR 4–10). The median PCI score was 14 (IQR 8–21).

**Table 2:** Baseline characteristics and univariate analysis in the derivation cohort

Variable	Entire cohort [*n* = 1068]	Univariate analysis in the derivation set [*n* = 796]		
		No readmission [*n* = 516]	Readmitted [*n* = 280]	*p*-Value
*Patient characteristics*				
Age, years	56 (47–64)	55 (47–63)	57 (47–65)	0.22
Male	525 (49%)	266 (52%)	134 (48%)	0.32
BMI, kg/m^2^	26.9 (23.7–31.2)	26.8 (23.6–31.0)	26.9 (23.9–31.4)	0.32
AA-CCI	6 (2–8)	6 (2–8)	4 (2–8)	0.09
Active smoking	77 (7.2%)	52 (8%)	17 (6%)	0.25
Repeat CRS/HIPEC	138 (13%)	66 (13%)	42 (15%)	0.39
ASA physical status				0.62
1	1 (0.1%)	1 (0.2%)	–	
2	216 (20%)	111 (22%)	55 (20%)	
3	744 (70%)	352 (68%)	201 (72%)	
4	107 (10%)	51 (10%)	24 (8%)	
5	1 (0.1%)	1 (0.2%)	–	
*Oncologic factors*				
Primary histology				0.15
Appendix	488 (46%)	240 (47%)	121 (43%)	
Well-differentiated	253 (52%)	135 (71%)	55 (29%)	
Moderately differentiated	156 (32%)	68 (64%)	39 (36%)	
Poorly differentiated	77 (16%)	37 (59%)	26 (41%)	
Colorectal	309 (29%)	141 (27%)	91 (32%)	
Peritoneal mesothelioma	113 (11%)	59 (11%)	22 (8%)	
Ovarian	57 (5%)	25 (5%)	21 (8%)	
Other	101 (9%)	51 (10%)	25 (9%)	
ECOG status				0.20
0	367 (34%)	190 (37%)	88 (32%)	
1	683 (64%)	320 (62%)	186 (66%)	
≥2	18 (2%)	6 (1%)	6 (2%)	
Preoperative chemotherapy	642 (60%)	301 (58%)	195 (70%)	0.002
Number of cycles	6 (5–10)	6 (5–9)	6 (5–10)	0.49
*Operative factors*				
PSS				0.85
0	62 (15%)	77 (15%)	37 (13%)	
1	314 (30%)	147 (29%)	87 (32%)	
2	356 (34%)	171 (34%)	91 (33%)	
3	220 (21%)	114 (22%)	61 (20%)	
PCI	14 (8–21)	13 (8–20)	15 (9–23)	0.004
OR time, hours	8.3 (6.8–10.3)	8.1 (6.6–10.0)	8.8 (7.2–10.9)	< 0.001
Estimated blood loss, mL	500 (250–900)	500 (250–800)	500 (300–900)	0.31
Intraoperative transfusion	231 (22%)	103 (20%)	69 (25%)	0.13
Number of visceral resections	2 (1–4)	2 (1–4)	3 (2–4)	< 0.001
Number of anastomoses	1 (0–2)	1 (0–2)	1 (1–2)	0.001
CC score				0.21
0	786 (74%)	389 (75%)	195 (70%)	
1	240 (22%)	107 (21%)	73 (26%)	
2+	39 (4%)	20 (4%)	11 (4%)	
*Hospitalization factors*				
Length of stay, days	12 (9–17)	11 (9–15)	13 (10–20)	<0.001
In-hospital complication	819 (76.7%)	380 (74%)	231 (83%)	0.005
CCI score	22.6 (8.7–36.2)	20.9 (0–33.5)	29.6 (20.9–45.6)	<0.001
Major complication	247 (23%)	100 (19%)	92 (33%)	<0.001
Leak diagnosed in hospital	103 (10%)	37 (7%)	37 (13%)	0.006
VTE event in hospital	66 (6%)	22 (4%)	27 (10%)	0.003
Postoperative transfusion	503 (47%)	218 (42%)	164 (59%)	<0.001
Percutaneous drain	208 (20%)	87 (17%)	74 (26%)	0.001
In-hospital TPN	190 (18%)	82 (16%)	63 (23%)	0.02
Return to ICU	114 (11%)	39 (8%)	42 (15%)	0.001
Return to OR	91 (8.5%)	27 (5%)	37 (13%)	<0.001
*Discharge factors*				
Discharge with ostomy	406 (38.0%)	165 (32%)	135 (48%)	< 0.001
Ileostomy	370 (91.1%)	153 (93%)	122 (90%)	
Colostomy	36 (8.9%)	12 (7%)	13 (10%)	
Day-of-discharge hemoglobin	8.9 (8.3–9.9)	9.1 (8.3–10.0)	8.8 (8.1–9.8)	0.005
Day-of-discharge white blood cell count	7.3 (5.0–10.4)	7.0 (4.9–9.9)	7.9 (5.4–11.0)	0.001
Day-of-discharge sodium	136 (134–138)	136 (134–138)	135 (133–138)	0.003
Discharge on antibiotics	197 (18.5%)	80 (16%)	68 (24%)	0.003
Discharge on therapeutic anticoagulation	121 (11.3%)	50 (10%)	38 (14%)	0.10
Discharge on regular diet	991 (92.8%)	489 (95%)	250 (89%)	0.005
Discharge with TPN	90 (8.4%)	36 (7%)	35 (13%)	0.01
Number of discharge medications	8 (5–10)	7 (5–10)	8 (6–12)	< 0.001
Discharge to a skilled nursing facility	86 (8.1%)	24 (5%)	36 (13%)	< 0.001

Data are displayed as mean (SD), median (IQR), or count (%)

*AA-CCI* age-adjusted Charlson–Deyo Comorbidity Index, *ASA* American Society of Anesthesiologists, *PSS* prior surgical score, *PCI* Peritoneal Cancer Index, *CC* completeness of cytoreduction, *CCI* Comprehensive Complication Index, *BMI* body mass index, *IQR* interquartile range, *CRS* cytoreductive surgery, *HIPEC* hyperthermic intraperitoneal chemotherapy, *ECOG* Eastern Cooperative Oncology Group, *OR* operating room, *VTE* venous thromboembolism, *TPN* total parenteral nutrition, *ICU* intensive care unit, *SD* standard deviation

### Predicting 90-Day Readmission

Using backwards elimination, seven factors that were associated with readmission were identified in the final multivariate model (*p* < 0.05). The final predictors in the model are listed in Table 3. This model was converted to a seven-component, 10-point score based on stoma creation during operation, PCI score ≥ 15, sodium level at discharge < 135 mEq/L, major postoperative complications, preoperative chemotherapy, hemoglobin level on discharge < 8 g/dL, and discharge to a skilled nursing facility.

**Table 3:** Derivation of model and score

Variable	OR	95% CI	*β* coefficient	*p*-Value	Point value
Stoma	1.41	1.02–1.96	0.345	0.04	1
PCI ≥15	1.43	1.04–1.96	0.358	0.03	1
Sodium < 135 mEq/L^a^	1.51	1.10–2.08	0.412	0.01	1
Major complication	1.54	1.07–2.20	0.430	0.02	1
Preoperative chemotherapy	1.63	1.18–2.26	0.488	0.00	1
Hemoglobin < 8 g/dL^a^	1.78	1.18–2.69	0.579	0.01	2
Discharge to SNF	2.63	1.48–4.67	0.967	0.00	3
				Total	10

^a^Laboratory values taken on the day of discharge

*PCI* Peritoneal Cancer Index, *SNF* skilled nursing facility, *OR* odds ratio, *CI* confidence interval

**Fig. 2:**
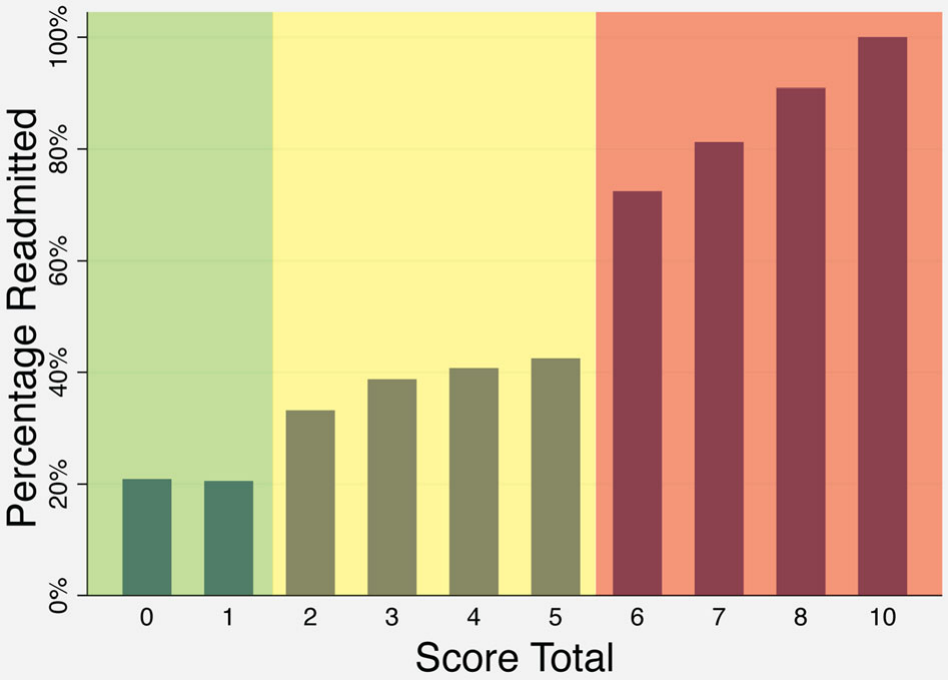
Percentage of patients readmitted in the derivation cohort, by score total. Patients were divided into low (0–2), moderate (2–5), and high-risk (6+) cohorts, based on the rate of 90-day readmission

Using this score, patients were stratified into low- (0–1), moderate- (2–5), and high-risk (6+) cohorts (Fig. 2). Low-risk patients in the derivation cohort had a predicted risk for readmission of 21.4% and an observed rate of readmission of 21.0%. High-risk patients in the derivation cohort had a predicted risk of readmission of 69.3% and an observed rate of readmission of 79.0%. The results were similar in the validation cohort (Table 4, electronic supplementary Tables 2–4).

**Table 4:** Observed versus predicted readmission

Points	Risk category	Number of patients (%)	Observed rate of readmission (%)	Predicted rate of readmission (%)
*Derivation cohort [n = 796]*				
0–1	Low	247 (31.0)	20.7	21.4
2–5	Moderate	492 (61.8)	37.4	38.1
6+	High	57 (7.2)	79.0	69.3
*Validation cohort [n = 272]*				
0–1	Low	91 (33.4)	17.6	21.4
2–5	Moderate	156 (57.4)	41.0	38.1
6+	High	25 (9.2)	76.0	69.3

Hosmer–Lemeshow goodness-of-fit statistics were *p* = 0.34 and *p* = 0.77 in the derivation and validation cohorts, respectively, indicating good calibration. The discriminatory power of the score was fair, with an AUC of 0.66 in the derivation cohort and 0.70 in the validation cohort. When applied to the whole cohort, the discrimination remained fair, with a c-statistic of 0.68.

### Surgical Quality Care Readmissions

We next examined our model performance in predicting 90-day readmissions related to quality of surgical care. In the derivation cohort, the model showed fair discrimination, with an AUC of 0.70 and good calibration with Hosmer–Lemeshow goodness-of-fit statistics (*p* = 0.47). In the validation cohort, the model had fair discrimination, with an AUC of 0.72 and good calibration with Hosmer–Lemeshow goodness-of-fit statistics (*p* = 0.87). These suggest the model is functional not only for predicting general CRS/HIPEC readmissions but also in targeting potentially preventable, surgical quality-related readmissions.

### Analysis of Full Model Performance versus Individual Factors

The discriminatory power of the full model was assessed in the validation cohort and compared with the performance of individual factors. The full model demonstrated the highest AUC (0.70) and lowest Brier score (0.200), indicating superior discrimination and calibration compared with individual elements.

## Discussion

In this study of 1068 encounters for CRS/HIPEC, we developed and internally validated a risk prediction tool for 90-day readmission following surgery. We found that at the time of discharge, seven factors easily and accurately stratified risk for readmission: stoma creation, PCI score, hyponatremia, major complications, preoperative chemotherapy, anemia, and discharge to a nursing facility. Our tool demonstrated good calibration and fair discrimination, suggesting that even within a high-risk population, it is possible to stratify risk for readmission to target a higher risk cohort for intervention.

These parameters are objective and are readily available at the time of discharge. Using this tool, 61% of our total CRS/HIPEC population was classified as moderate risk for readmission (scores of 2–5), with a predicted rate of readmission of 38% and an observed rate of 38%. An additional 8% were classified as high risk for readmission (scores 6+), with a predicted rate of readmission of 69% and an observed rate of readmission of 78%. A similar performance in predicting overall readmissions and surgical quality readmissions suggests this score could be clinically applicable in targeting preventable readmissions.^11^

At present, the discharge process at our institution is not protocolized. All CRS/HIPEC patients receive a 30-day supply of prophylactic anticoagulation and routine home health care; however, time to postoperative follow-up, telephone and telemedicine check-ins, and other services are variable. Ideally, patients would receive targeted follow-up and specific interventions tailored to their needs and relative risk for readmission. Identifying which patients are most at risk and likely to benefit from intervention is a crucial first step in improving readmission rates.

Drivers of readmission following surgery are complex and varied. As a consequence, published interventions to prevent readmission are diverse. In a systematic review examining readmissions for medical and surgical patients, Leppin and colleagues found an overall reduction in the risk of readmission of 18% associated with readmission reduction programs across the 42 examined studies.^18^ The authors suggest that multimodal interventions focused on coordinating care and increasing patients’ ability to engage in self-care postdischarge were most effective at reducing readmissions. Similarly, Jones et al. examined transitional care interventions after surgery and found that coordinated discharge planning, patient education, and protocolized follow-up reduced readmissions.^19^ Evaluated programs demonstrated reductions in readmissions ranging from 8 to 24%. In both reviews, the authors stressed that successful programs offered comprehensive predischarge assessment of needs, together with coordinated discharge and tailored postdischarge follow-up.

We propose to use our score to develop and implement an evidence-driven quality improvement program to address readmission following CRS/HIPEC at our institution. Theoretical application of our score, utilizing a cut-off of 2 and above to target patients for readmission intervention, would target 68% of our cohort and 82% of all readmitted patients, with a sensitivity of 82%, specificity of 39%, and AUC of 0.61 (electronic supplementary Table 6). We plan to target moderate- and high-risk patients through tailored advanced discharge planning addressing nutritional needs, fluid status, pain management, and wound/stoma care, as well as graduated advance practice provider telemedicine and in-person follow-up based on risk score.

Our overall readmission rate of 35.5% is consistent with prior reports, however notably somewhat higher than other high-volume centers.^1^,^3^ As other CRS/HIPEC readmission studies have demonstrated, we found complications were predictive of readmission.^12^–^14^ Our model derivation included models with both composite measures of complications (CCI total, major complications) and individual complications (VTE, leak, reoperation). Ultimately, in multivariate analysis, the presence of a major complication remained a strong predictor of readmission.

Several measures of operative complexity and specific procedures were examined for association with readmissions. While many measures, including operative length, blood loss, number of resections, and number of anastomoses, were significant on univariate analysis, stoma creation, which included both ileostomy and colostomy creation, remained significant on multivariate analysis. Ostomies are recognized risk factors for readmission following CRS/HIPEC and general colorectal surgery.^3^,^13^,^20^ Stoma creation may be a surrogate marker of more complex surgery. Additionally, stoma creation, particularly among elderly patients, places patients at higher risk for fluid and electrolyte issues, a common indication for readmission in our cohort.^20^

In our final model, higher PCI score was associated with readmission. This is notable in that previous studies have suggested PCI scores were not independently associated with readmission. In their examination of 223 patients undergoing CRS/HIPEC, Dreznik and colleagues^14^ found no difference in PCI score between patients with and without readmission (mean PCI 12.6 vs. 12.4, *p* = 0.53). Lee and colleagues^3^ conducted a multi-institutional review of readmissions at 12 high-volume CRS/HIPEC centers, and, in their study of 2017 cases, they examined preoperative and operative predictors of readmission. There was a trend toward higher PCI scores in the readmitted cohort (15 vs. 13, *p* = 0.07); however, on multivariate analysis, PCI scores above 20 were not associated with readmission (*p* = 0.21). Our population had overall higher PCI scores, potentially signifying higher preoperative disease burden and accounting for differences between this and prior studies. Additionally, our model included postoperative and day-of-discharge variables not included in the analysis by Lee et al., which may underlie model differences.

Pre-CRS/HIPEC chemotherapy emerged as a significant predictor of readmission. In their examination of readmissions, Lee et al. found neoadjuvant chemotherapy to be associated with readmission on univariate analysis (*p* < 0.01), however in their ultimate multivariate model, it did not remain a significant predictor (*p* = 0.59).^3^ Notably, preoperative chemotherapy rates were significantly higher in our cohort (60%), with relatively high preoperative chemotherapy burden (six cycles, IQR 5–10). This higher burden and duration of chemotherapy may account for differences in significance.

Day-of-discharge laboratory studies, hemoglobin and sodium, were also significant predictors of readmission; both have previously been found to be significant predictors of readmission. Merkow et al. examined nearly 500,000 patients undergoing surgery in the US and found that complications due to bleeding or anemia were the third most common cause of readmission.^21^ In their analysis of readmissions following CRS/HIPEC, Martin et al. found perioperative transfusion was associated with readmission at 30 days.^12^ In our analysis, while perioperative transfusion was predictive of readmission by univariate analysis, only anemia on the day of discharge remained significant on multivariate analysis. Similarly, hyponatremia was found to be significantly associated with readmission in our final analysis. Hyponatremia features in several common risk prediction models, such as the HOSPITAL and MELD-Na scores, and has been independently associated with increased complications and mortality in cardiac surgery patients.^10^,^22^,^23^ Underlying mechanisms for this association remain unclear, however hyponatremia has been identified as a marker of systemic inflammation and associated infection.^24^ Given the high rates of infection among CRS/HIPEC readmissions, these patients may be showing early signs at the time of discharge. Alternatively, these laboratory values may function more as general markers of prognosis and comorbidities, with sodium reflecting protein malnutrition and anemia signifying marrow suppression due to preoperative or intraoperative chemotherapy.

Finally, discharge to a skilled nursing facility emerged as the highest weighted factor within our model despite not being associated with readmission in previous studies.^3^,^13^ Notably, a significantly higher portion of our population, i.e. 8% of our cohort, was discharged to a nursing facility following index hospitalization. Among discharges examined by Lee et al. and Kelly et al., 2.7% and 3.1% of patients were discharged to a nursing home, respectively. It is possible that in our model, discharge to a nursing facility stands in for potentially unmeasured differences in physical status or frailty, accounting for differences in significance between studies.

This study has several notable limitations. Although we examined readmissions out to 90 days, including both index and non-index hospitalizations, it is possible that additional patients were readmitted to non-index facilities without our knowledge. However, our similar readmission rates with those previously reported suggested that the portion of missed readmissions is likely small, or at least on par with previous studies.^3^ Therefore, we do not feel there is sufficient evidence to suggest this portion of missed readmissions would significantly alter our findings. Additionally, although we excluded in-hospital mortalities and discharges to hospice, there is the potential that patients died after discharge without readmission. We examined 90-day survival after discharge within our cohort, with mortalities verified by the National Death Index; 26 patients (2.4%) died within 90 days of discharge. All of these patients were readmitted first, suggesting that the potential influence of missed mortalities would be low. Finally, this study only performed an internal validation of our model and is drawn from the experience of a single, high-volume center. Limited numbers of patients with scores above 8 impairs calibration and estimation. Both prospective and external validation is necessary to further refine our stratification and improve our risk score.

This study represents a novel risk prediction tool in a high-risk population. In similar prediction models, diagnosis with malignancy or disseminated cancer is heavily weighted towards readmission, making such tools less useful when addressing a surgical oncology population. In this study, we show that even among patients with baseline high risk for readmission, we can stratify risk to target future efforts to the most vulnerable cohort in an effort to improve patient outcomes.

## Conclusions

In this analysis, we derive and validate a practical prediction model to assess risk for 90-day readmission in patients undergoing CRS/HIPEC. To our knowledge, this represents the first risk prediction tool specifically generated for this complex population. We propose to use this score to target moderate and high-risk patients for enhanced discharge planning and intensive postdischarge monitoring and follow-up.

## Electronic supplementary material

Below is the link to the electronic supplementary material.

Supplementary material 1 (DOCX 31 kb)
