# Supplementary material for: Predischarge Prediction of Readmission After Cytoreductive Surgery and Hyperthermic Intraperitoneal Chemotherapy: Derivation and Validation of a Risk Prediction Score
Source: Ann Surg Oncol. 2021 Jan 23;28(9):5287–96. doi: 10.1245/s10434-020-09547-7 (PMC8349345; doi:10.1245/s10434-020-09547-7)
Supplement: Supplementary file 1 — Supplementary material 1 (DOCX 31 kb) [file 10434_2020_9547_MOESM1_ESM.docx]

**Supplemental Tables**

| **Supplemental Table 1:** Missing Data for Predictor Variables | | | | | | |
| --- | --- | --- | --- | --- | --- | --- |
|  | **No Readmission** | | **Readmission** | | **Total** | |
| Number of Patients, total | **N=689** | | **N=379** | | **N=1068** | |
| Missing Variable, number (%) | | | | | | |
| **Patient Characteristics** | | | | | | |
| Age | 0 | 0% | 0 | 0% | 0 | 0% |
| Male | 0 | 0% | 0 | 0% | 0 | 0% |
| BMI | 4 | 0.6% | 1 | 0% | 5 | 0.5% |
| AA-CCI | 0 | 0% | 0 | 0% | 0 | 0% |
| Active Smoking | 0 | 0% | 0 | 0% | 0 | 0% |
| Repeat CRS HIPEC | 0 | 0% | 0 | 0% | 0 | 0% |
| ASA Physical Status | 0 | 0% | 0 | 0% | 0 | 0% |
| **Oncologic Factors** | | | | | | |
| Primary Histology | 0 | 0% | 0 | 0% | 0 | 0% |
| ECOG Status | 0 | 0% | 0 | 0% | 0 | 0% |
| Preoperative Chemotherapy | 0 | 0% | 0 | 0% | 0 | 0% |
| Number of Cycles | 114 | 29% | 65 | 25% | 179 | 28% |
| **Operative Factors** | | | | | | |
| PSS | 9 | 1% | 6 | 2% | 15 | 1% |
| PCI | 1 | 0.1% | 6 | 2% | 7 | 0.7% |
| OR Time | 0 | 0% | 0 | 0% | 0 | 0% |
| Estimated Blood Loss | 6 | 0.9% | 1 | 0.3% | 7 | 0.7% |
| Intraoperative Transfusion | 0 | 0% | 0 | 0% | 0 | 0% |
| Number of visceral resections | 0 | 0% | 0 | 0% | 0 | 0% |
| Number of Anastomoses | 0 | 0% | 0 | 0% | 0 | 0% |
| **Hospitalization Factors** | | | | | | |
| Length of Stay | 0 | 0% | 0 | 0% | 0 | 0% |
| In Hospital Complication | 0 | 0% | 0 | 0% | 0 | 0% |
| CCI, | 0 | 0% | 0 | 0% | 0 | 0% |
| Major Complication | 0 | 0% | 0 | 0% | 0 | 0% |
| Leak Diagnosed in Hospital | 0 | 0% | 0 | 0% | 0 | 0% |
| VTE Event in Hospital | 0 | 0% | 0 | 0% | 0 | 0% |
| Postoperative Transfusion | 0 | 0% | 0 | 0% | 0 | 0% |
| Percutaneous Drain | 0 | 0% | 0 | 0% | 0 | 0% |
| In hospital TPN | 0 | 0% | 0 | 0% | 0 | 0% |
| Return to ICU | 0 | 0% | 0 | 0% | 0 | 0% |
| Return to OR | 1 | 0.1% | 0 | 0% | 1 | 0.1% |
| **Discharge Factors** | | | | | | |
| Discharge with ostomy | 0 | 0% | 0 | 0% | 0 | 0% |
| Day of discharge Hemoglobin | 6 | 0.9% | 2 | 0.5% | 8 | 0.7% |
| Day of discharge White blood cell count | 7 | 1% | 1 | 0.3% | 8 | 0.7% |
| Day of discharge Sodium | 7 | 1% | 1 | 0.3% | 8 | 0.7% |
| Discharge on Antibiotics | 0 | 0% | 0 | 0% | 0 | 0% |
| Discharge on Anticoagulation | 0 | 0% | 0 | 0% | 0 | 0% |
| Discharge with TPN | 0 | 0% | 0 | 0% | 0 | 0% |
| Number of discharge meds | 24 | 3% | 0 | 0% | 24 | 2% |
| Discharge disposition to skilled nursing facility | 0 | 0% | 0 | 0% | 0 | 0% |

| **Supplemental Table 2:** Scoring System Details in the Derivation Cohort | | | | | |
| --- | --- | --- | --- | --- | --- |
| **Score Total** | **Number of Patients** | **Number of Patients Readmitted** | **Observed Readmission (%)** | **Predicted Readmission (%)** | **O/E Ratio** |
| 0 | 67 | 14 | 20.9% | 17.2% | 1.21 |
| 1 | 180 | 37 | 20.6% | 22.9% | 0.90 |
| 2 | 184 | 61 | 33.2% | 29.8% | 1.11 |
| 3 | 160 | 62 | 38.8% | 37.8% | 1.03 |
| 4 | 108 | 44 | 40.7% | 46.5% | 0.88 |
| 5 | 40 | 17 | 42.5% | 55.3% | 0.77 |
| 6 | 29 | 21 | 72.4% | 63.9% | 1.13 |
| 7 | 16 | 13 | 81.3% | 71.7% | 1.13 |
| 8 | 11 | 10 | 90.9% | 78.3% | 1.16 |
| 9 | - | - | - | 83.8% | - |
| 10 | 1 | 1 | 100.0% | 88.1% | 1.14 |

| **Supplemental Table 3**: Scoring System Details in the Validation Cohort | | | | | |
| --- | --- | --- | --- | --- | --- |
| **Score Total** | **Number of Patients** | **Number of Patients Readmitted** | **Observed Readmission (%)** | **Predicted Readmission (%)** | **O/E Ratio** |
| 0 | 24 | 3 | 12.5% | 17.2% | 0.73 |
| 1 | 67 | 13 | 19.4% | 22.9% | 0.85 |
| 2 | 53 | 21 | 39.6% | 29.8% | 1.33 |
| 3 | 50 | 21 | 42.0% | 37.8% | 1.11 |
| 4 | 36 | 16 | 44.4% | 46.5% | 0.96 |
| 5 | 17 | 6 | 35.3% | 55.3% | 0.64 |
| 6 | 16 | 12 | 75.0% | 63.9% | 1.17 |
| 7 | 5 | 3 | 60.0% | 71.7% | 0.84 |
| 8 | 4 | 4 | 100.0% | 78.3% | 1.28 |
| 9 | - | - | - | 83.8% | - |
| 10 | - | - | - | 88.1% | - |

| **Supplemental Table 4**: Scoring System Details in Whole Cohort | | | | | |
| --- | --- | --- | --- | --- | --- |
| **Score Total** | **Number of Patients** | **Number of Patients Readmitted** | **Observed Readmission (%)** | **Predicted Readmission (%)** | **O/E Ratio** |
| 0 | 91 | 17 | 18.7% | 17.2% | 1.09 |
| 1 | 247 | 50 | 20.2% | 22.9% | 0.88 |
| 2 | 237 | 82 | 34.6% | 29.8% | 1.16 |
| 3 | 210 | 83 | 39.5% | 37.8% | 1.05 |
| 4 | 144 | 60 | 41.7% | 46.5% | 0.90 |
| 5 | 57 | 23 | 40.4% | 55.3% | 0.73 |
| 6 | 45 | 33 | 73.3% | 63.9% | 1.15 |
| 7 | 21 | 16 | 76.2% | 71.7% | 1.06 |
| 8 | 15 | 14 | 93.3% | 78.3% | 1.19 |
| 9 | - | - | - | 83.8% | - |
| 10 | 1 | 1 | 100.0% | 88.1% | 1.14 |

| **Supplemental Table 5:** Evaluation of Model Discrimination Versus Individual Factors | | | | |
| --- | --- | --- | --- | --- |
| **Variable** | **AUC** | **95% CI** | **Brier Score** | **P Value** |
| Full Model | 0.7 | 0.63-0.76 | 0.2003 | 0.489 |
| CCI Total | 0.66 | 0.60-0.73 | 0.2140 | 0.471 |
| Discharge to SNF | 0.57 | 0.53-0.61 | 0.2201 | 0.5 |
| Hemoglobin <8 | 0.5 | 0.46-0.55 | 0.2315 | 0.5 |
| Major Complication | 0.56 | 0.50-0.61 | 0.2274 | 0.5 |
| Number of Resections | 0.59 | 0.52-0.66 | 0.2257 | 0.499 |
| PCI Score | 0.59 | 0.52-0.66 | 0.2285 | 0.487 |
| Preoperative Chemotherapy | 0.55 | 0.49-0.62 | 0.2289 | 0.497 |
| Stoma | 0.65 | 0.59-0.71 | 0.2122 | 0.459 |
| Sodium <135 | 0.57 | 0.51-0.63 | 0.2267 | 0.489 |

| **Supplemental Table 6**: Evaluation of Potential Thresholds for Intervention | | | | | | |
| --- | --- | --- | --- | --- | --- | --- |
| **Score Cutoff Evaluated** | **n (%)** | **AUC** | **Sensitivity** | **Specificity** | **Positive Predictive Value** | **Correct Classification** |
| 1 | 977 (91.5%) | 0.53 | 95.5% | 10.7% | 37.1% | 81.3% |
| 2 | 730 (68.4%) | 0.61 | 82.3% | 39.3% | 42.7% | 80.2% |
| 3 | 493 (46.2%) | 0.61 | 60.7% | 61.8% | 46.7% | 74.1% |
| 4 | 283 (26.5%) | 0.6 | 38.8% | 80.3% | 51.9% | 70.5% |
| 5 | 139 (13.0%) | 0.58 | 23.0% | 92.5% | 62.6% | 68.6% |
| 6 | 82 (7.7%) | 0.57 | 16.9% | 97.4% | 78.1% | 68.1% |
| 7 | 37 (3.5%) | 0.54 | 8.2% | 99.1% | 83.8% | 66.3% |
| 8 | 16 (1.5%) | 0.52 | 4.0% | 99.9% | 93.8% | 65.4% |
